# An Unusual Cause of Cardiac Tamponade during Cardiac Catheterization Study

**DOI:** 10.1155/2014/652592

**Published:** 2014-09-18

**Authors:** Deepanwita Das, Monalisa Datta, Somnath Dey, Jyotiranjan Parida, Rupesh Kumar, Arindam Pande

**Affiliations:** ^1^Department of Cardiac Anaesthesia, Institute of Postgraduate Medical Education & Research, SSKM Hospital, Kolkata, India; ^2^Department of Cardiothoracic Surgery, Institute of Postgraduate Medical Education & Research, SSKM Hospital, Kolkata, India; ^3^Department of Cardiology, Institute of Postgraduate Medical Education & Research, SSKM Hospital, Kolkata, India

## Abstract

*Introduction.* Catheter-based diagnostic and therapeutic procedures are rapidly advancing. However, catheter related complications sometimes become life threatening. Cardiac tamponade is a rare but serious complication of this procedure. We have summarized one cardiac tamponade pejoration due to secondary coronary vessels laceration by the implanted pericardial drainage. *Case report*. A 4-year-old baby having Tetralogy of Fallot was posted for diagnostic catheterization study. Patient was induced with sevoflurane and spontaneous respiration was maintained. After catheter insertion to RV, dye was injected through the catheter which rapidly spread into the pericardial cavity indicating right ventricle perforation. Immediately, blood was aspirated under transthoracic echocardiographic guidance and hemodynamics started improving. For the provision of quick access to aspirate further collection, an intrapericardial sheath was inserted after multiple attempts. Patient's condition started deteriorating again. TTE revealed again some collection and it was increasing gradually. On exploration, it was found that there was continuous bleeding from a lacerated epicardial vessel which contributed to the pericardial collection leading to further tamponade effect. This second iatrogenic injury complicated the management of the first iatrogenic cardiac perforation and, thereby, created a life-threatening situation which needed immediate surgical exploration. *Discussion*. Usual cause of tamponade after right ventricular perforation is bleeding from the RV, but in our case the second tamponade was not due to bleeding from the RV, but was rather from new laceration injury of epicardial vessels which was remained undiagnosed till exploration.

## 1. Introduction

In the era of interventional cardiology, catheter-based diagnostic and therapeutic procedures are rapidly advancing. However, catheter related complications are still there [1]. It occurs more frequently with debulking devices and often as a consequence of guide wire migration and injury [2]. Acute hemorrhagic pericardial collection secondary to iatrogenic inadvertent cardiac perforation often leads to fatal tamponade. Echo-guided pericardiocentesis has been shown to be effective and primary management [3], although it is not always safe. Multiple attempts of pericardiocentesis are dangerous. Vigorous attempts to aspirate can also cause further injury to cardiac vessels that aggravates the tamponade effect.

Recently we have encountered a catastrophic hemorrhagic tamponade which leads to severe hemodynamic instability while managing a primary iatrogenic right ventricular perforation during catheterization study. The case has been effectively managed by early diagnosis and proper management of catastrophic sequela of pericardiocentesis.

## 2. Case Report

A 4-year-old baby weighing 10 kg, having Tetralogy of Fallot, was posted for diagnostic catheterization study. On examination, there was central cyanosis, grade IV clubbing, pulse rate 110/min, NIBP 80/60 mmHg, ejection systolic murmur over pulmonary area (Table 1). In chest X ray, there was cardiomegaly and boot shaped heart with oligemic lung fields. SpO_2_ in room air was 86%.

In the operating room, intravenous cannula was put after prior application of prilox patch. All ASA standard monitors were attached. Premedication was given with glycopyrrolate (0.1 mg), fentanyl (20 mcg), and midazolam (1 mg) intravenously 5 minutes before induction. Induction was done with sevoflurane (6%) and LMA (size 2) was inserted. Patient was kept in spontaneous ventilation. Local infiltration with 1% lignocaine was given and femoral artery and femoral vein were cannulated. Guide wire was introduced, over which NIH catheter was inserted to RV. At that time, ECG showed different types of ill-sustained arrhythmia, although blood pressure was stable. When dye was injected through the catheter, it rapidly started spreading into the pericardial cavity which can be clearly seen in fluoroscopy. There was perforation of RV and dye mixed blood extravasated to pericardium. NIBP suddenly dropped to 40/30 mmHg and heart rate decreased (50/min). Inj atropine (0.2 mg) was administered immediately and patient was resuscitated with intravenous fluids. But, unfortunately, the arrhythmia continued and ultimately there was cardiac arrest. Patient was intubated immediately and cardiopulmonary resuscitation (CPR) was started. Transthoracic echocardiography showed cardiac tamponade with massive pericardial blood collection. Immediately pericardiocentesis was attempted under the guidance of echocardiography and 150 mL of blood was aspirated. Fortunately, heart started to contract again, but hypotension still persists. Blood transfusion was started and patient's vitals were improved. ECG was showing sinus rhythm but, despite that, NIBP was low (60/42 mmHg). TTE was done again, but this time there was no further pericardial collection. We have waited for another 15 minutes to observe further accumulation of blood. But there was no further collection. Then, the cardiologist decided to put an intrapericardial sheath to have a provision for quick access to aspirate if further collection occurs. To introduce the sheath, a guide wire was attempted to insert once again, but it was unsuccessful. Guide wire was obstructed somewhere and not easily passed into. Multiple attempts were needed under fluoroscopic guidance to insert the sheath.

Patient's condition started deteriorating again, with NIBP 40/30 mmHg and HR 140/min. TTE revealed again some collection and it was increasing gradually. Fifty millilitres of blood was aspirated out immediately. It was decided to call up the cardiothoracic surgeons immediately for sternotomy and exploration under direct vision, keeping cardiopulmonary bypass circuit as a standby. Femoral arterial line and central venous line were inserted. IBP was 40/30 mmHg. CVP was found to be 15 mmHg. Inotropic (dopamine and adrenaline) support was started. After exploration by midline sternotomy, about 80 mL blood drained out. Surprisingly it was found that the perforated site of RV has stopped bleeding spontaneously by the hypertrophied RV muscle mass effect, but there was continuous bleeding from a lacerated epicardial vessel which contributed to the pericardial collection leading to further tamponade effect.

This new epicardial vessel injury was caused by the multiple attempts to insert guide wire for introduction of the sheath. This second iatrogenic injury complicated the management of the first iatrogenic cardiac perforation and, thereby, created a life-threatening situation which needed immediate surgical exploration. Pericardial pledged repair of the epicardial vessel was done by the surgeons. One unit of packed red cells was transfused intraoperatively. Intraoperative ABG showed metabolic acidosis, which was corrected immediately. Patient was stable hemodynamically with IBP 90/70 mmHg, heart rate 112/min, and ECG in sinus rhythm. Patient was extubated on table and shifted to ICU with continuous monitoring of all vitals. Postoperative course was uneventful. On the third day baby was shifted to ward with stable hemodynamics without any inotropic support.

## 3. Discussion

Tetralogy of Fallot is a cardiac anomaly that refers to a combination of four related cardiac defects that commonly occur together. The four defects are ventricular septal defect, overriding of aorta, right ventricular outflow tract obstruction, and right ventricular hypertrophy. In 2%–14% of patients with TOF, there are associated coronary artery anomalies [4]. Diagnostic catheterization study before surgical correction is a usual procedure but is not free from complication. Cardiac perforation is a rare but life-threatening complication of catheterization. The incidence of cardiac perforation has been reported to be 1.5% to 4.7% for valvuloplasty [5, 6], 0.2% to 1% for radiofrequency ablation [7, 8], 0.1% to 0.2% for electrophysiologic study [9], 0.5% for cardiac biopsy [10], 0.03% for coronary angioplasty [11], and 0.01% for diagnostic catheterization [5]. Iatrogenic cardiac perforation often ends in life-threatening situation, as immediate surgical exploration is the only way to save the patient. In the present echo era, there is the novel opportunity to perform quick and effective echo-guided pericardiocentesis to manage such complications, thereby saving the patient. In our patient, the echo-guided aspiration was effective and hemodynamics improved markedly. The pericardial sheath was inserted in the pericardial cavity for better access for quick aspiration of subsequent collection if any.

Though echo-guided pericardiocentesis is the primary management of acute tamponade, still it is not free of complications. The epicardial vessel injury by the sharp metal tip guide wire is very rare but is a potential complication, as we have encountered in our case. Pericardiocentesis is needed very quickly within few seconds in a hemodynamically unstable patient and it is more challenging when the patient is a paediatric one. Paediatric patient has less cardiopulmonary reserve; moreover, small amount of acute collection can cause the tamponade to impair the contraction of the heart and cardiac output. The only aim of management is releasing the tamponade effect. The usual cause of tamponade after right ventricular perforation is bleeding from the RV, but in our case the second tamponade was not due to bleeding from the RV but was rather from new laceration injury of epicardial vessels which was undiagnosed till exploration. It is really very difficult to diagnose the exact site of bleeding by echocardiography. Prompt diagnosis and early treatment are very important in those situations. Multiple attempts are very dangerous and should always be avoided. Interventions must be steady and smooth in all cases, especially in paediatric ones as delay between diagnosis and resuscitation endangers the life of a baby. The essence of this case report is that primary complication of iatrogenic RV perforation was effectively managed by echo-guided pericardiocentesis but the iatrogenic epicardial vessel injury, which was a real challenge to diagnose echocardiographically, necessitates surgical management to save the patient.

The early diagnosis and prompt treatment as teamwork with cardiologists, cardiac anaesthesiologists, and cardiothoracic surgeons were the pearl for the great success.

## Figures and Tables

**Table 1:** Clinical parameters.

Parameters	Before pericardiocentesis	Shortly after pericardiocentesis	Before surgical revision
Heart rate (/min)	50	130	140
NIBP (mmHg)	40/30	60/42	40/30
SpO_2_ (%)	88	88	86
CVP (mmHg)	Not measured	Not measured	15
